# Understanding in an instant: Neurophysiological evidence for mechanistic language circuits in the brain

**DOI:** 10.1016/j.bandl.2008.12.001

**Published:** 2009-08

**Authors:** Friedemann Pulvermüller, Yury Shtyrov, Olaf Hauk

**Affiliations:** Medical Research Council, Cognition and Brain Sciences Unit, 15 Chaucer Road, Cambridge, UK

**Keywords:** EEG, Concept, Speech, Meaning, MEG, Neurophysiology, Spatio-temporal pattern of brain activity, Time course, Word recognition

## Abstract

How long does it take the human mind to grasp the idea when hearing or reading a sentence? Neurophysiological methods looking directly at the time course of brain activity indexes of comprehension are critical for finding the answer to this question. As the dominant cognitive approaches, models of serial/cascaded and parallel processing, make conflicting predictions on the time course of psycholinguistic information access, they can be tested using neurophysiological brain activation recorded in MEG and EEG experiments. Seriality and cascading of lexical, semantic and syntactic processes receives support from late (latency ∼1/2 s) sequential neurophysiological responses, especially N400 and P600. However, parallelism is substantiated by early near-simultaneous brain indexes of a range of psycholinguistic processes, up to the level of semantic access and context integration, emerging already 100–250 ms after critical stimulus information is present. Crucially, however, there are reliable latency differences of 20–50 ms between early cortical area activations reflecting lexical, semantic and syntactic processes, which are left unexplained by current serial and parallel brain models of language. We here offer a mechanistic model grounded in cortical nerve cell circuits that builds upon neuroanatomical and neurophysiological knowledge and explains both near-simultaneous activations and fine-grained delays. A key concept is that of discrete distributed cortical circuits with specific inter-area topographies. The full activation, or ignition, of specifically distributed binding circuits explains the near-simultaneity of early neurophysiological indexes of lexical, syntactic and semantic processing. Activity spreading within circuits determined by between-area conduction delays accounts for comprehension-related regional activation differences in the millisecond range.

## 1 Introduction

**Fig. 1:**
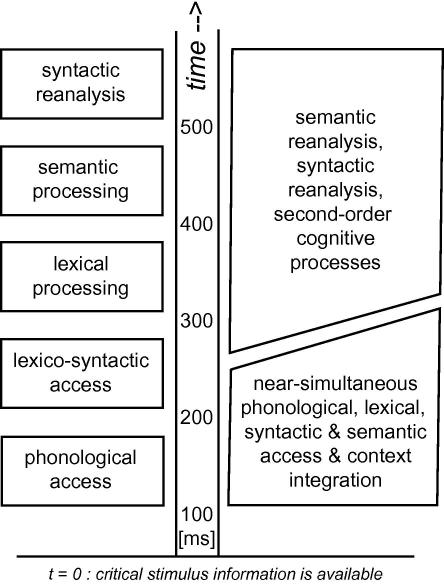
Modular seriality versus interactive parallelism. Object and language comprehension requires access to long-term memory traces in the brain. In one view, this access process is a chain of subprocesses starting sequentially, with process-onset-asynchronies of hundreds of milliseconds. One postulate has been that phonological and lexico-syntactic features of a word are accessed first (150 ms) later followed by semantic access and context integration (400 ms). Such serial or cascaded processing is sometimes attributed to separate processing subcomponents, modules, that each are envisaged to process independently their share of the input information (Fodor, 1983; Friederici, 2002; Morton, 1969; Shallice, 1988). A serial model with a time scale indexing the time range when after stimulus presentation component processes might occur in the brain, is given on the left. As an alternative to seriality, different types of information may be accessed in parallel in the perception and recognition process. Upon analysis of the physical features and form of a stimulus or symbol, processing of linguistic-conceptual information, for example phonological, lexical, syntactic and semantic information, does accordingly occur early and at roughly the same time. This idea of parallelism is sometimes connected with that of an interactive system allowing for free information exchange between processing subcomponents (Gaskell & Marslen-Wilson, 2002; Tanenhaus, Spivey-Knowlton, Eberhard, & Sedivy, 1995). A parallel model of psycholinguistic information access is shown on the right.

A major debate in cognitive science centers around the question whether different kinds of information extracted from a stimulus are processed in a serial fashion or rather in parallel. Serial and cascaded models posit that the onsets of these processes are sequential,^1^ whereas parallel models assume simultaneous information access (Fig. 1). Behavioral experiments capitalizing on reaction times and performance accuracy have not been conclusive in deciding between the two competing views, as available evidence could be interpreted in favor of both (Marslen-Wilson & Tyler, 1975; Posner & Pavese, 1998; Pulvermüller, 2007).

Electro- and magnetoencephalography (EEG and MEG) measure the brain response with millisecond temporal resolution. At any given time point the neurophysiological response is influenced only by processes that occurred at earlier latencies. Therefore, the latency of the earliest reliable neurophysiological correlate of a psycholinguistic variable provides an upper limit for the time range within which the corresponding type of information is accessed. One of the most intensely debated issues in psycholinguistics, at what latencies phonological, orthographic, lexical, syntactic and semantic information is first accessed, can therefore be addressed using neurophysiological means.

Most previous experiments have employed factorial experimental designs and subtraction logic: The average response across a number of items is computed for different stimulus categories (the “event-related potential”, ERP), and the difference between these averages is evaluated statistically. In order to draw conclusions on a specific psycholinguistic process in the brain (e.g. lexical access), stimulus groups should differ only on parameters that can be assumed to modulate this particular process (e.g. word frequency). Unfortunately, there is a range of perceptual and psycholinguistic properties of language stimuli that potentially affect the brain response, and they are strongly intercorrelated (see, for example, Hauk, Davis, Ford, Pulvermüller, & Marslen-Wilson, 2006). This poses particular challenges to stimulus selection, both in the investigation of early and late neurophysiological effects. Possibilities to address this problem include precise stimulus matching for a range of variables (see Pulvermüller, 1999, 2007), regression designs that control for several variables at a time (Hauk, Davis, Ford, Pulvermüller, & Marslen-Wilson, 2006a; Hauk et al., 2006b) and the presentation of identical stimulus materials (words, syllables, phonemes, letters) in different contexts (see, for example, Hagoort, Brown, & Groothusen, 1993; Penolazzi, Hauk, & Pulvermüller, 2007).

Here, we first review neurophysiological evidence for cognitive seriality. Neurophysiological indexes of lexico-syntactic, semantic and deep syntactic processes emerging in sequence (cf. Footnote 1) at about 200, 400 and 600 milliseconds, respectively (Section 2). We then review reports of near-simultaneous early indexes of lexical, syntactic and semantic processes at 100–250 ms in both written and spoken language processing and conclude that these results tend to weigh in favor of parallel processes (See Section 3). An interim summary is followed by a closer look at the *near* simultaneous early activations, which reveals significant millisecond differences (See Section 5). Their explanation requires a mechanism for sequencing at a much finer time scale than the ones offered by established serial models. We exploit a neurobiological model of language to explain reliable millisecond activation delays (Sections 6 and 7). Summary and Outlook sections conclude the review.

## 2 Late seriality

In this section, we will briefly review previous evidence based on “classical” ERP components for serial processing of semantic and syntactic information. Probably the most extensively studied ERP component related to language is the N400 (Kutas & Hillyard, 1980), which is maximal 1/3 to 1/2 of a second after onset of the critical stimulus word. Originally, the N400 was reported in response to words that appear in sentences where they do not fit semantically (“The fish swam in the *fee”)*. However, it has since been reported to be elicited by single words out of context as well (Holcomb & Neville, 1990).

**Fig. 2:**
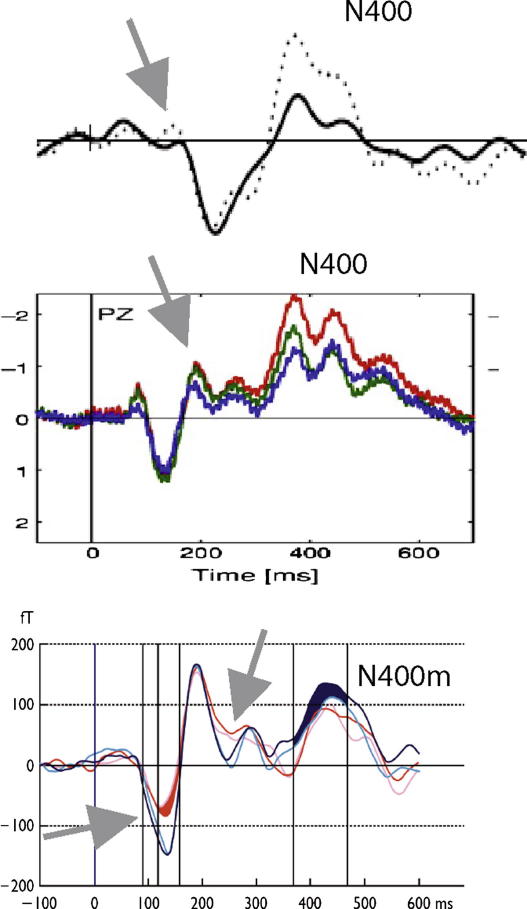
Electric and magnetic event-related brain response to rare and common words. Top diagram: In addition to a clear difference at ∼400 ms (N400), note also the small trend towards a dissociation at 100–200 ms. Solid lines indicate common words and broken lines rare ones (modified from Kutas & Federmeier, 2000). Middle diagram: Event-related potentials to rare (in red), moderately frequent (green) and highly frequent words (blue), again showing a large dissociation at ∼400 ms and a smaller one before and around 200 ms (modified from Dambacher et al., 2006). Bottom diagram: Magnetic brain response to short (in red) and long (in blue) words that occur rarely (in dark colors) or frequently (light colors) in language use (modified from Assadollahi & Pulvermüller, 2001). Note that the earliest effect appears specifically for short words. Around 200 ms, a significant frequency effect is present for all words and the late effect in the magnetic N400 is strongest for longer words. Arrows indicate early effects. The *x*-axes are aligned and give time in ms. The *y*-axes indicate magnitude of electric potentials (in μV, top and middle diagrams, negative is up) and magnetic fields (in fT, bottom diagram).

A range of psycholinguistic features have been found to be reflected in the N400. This component is larger to meaningless pseudowords than it is to words (Holcomb & Neville, 1990) and similarly larger to rare words than to common ones (Fig. 2, Kutas & Federmeier, 2000; Van Petten & Kutas, 1990). Furthermore, the N400 appears to be modulated by neighborhood size (Holcomb, Grainger, & O’Rourke, 2002), morphological family size (Pylkkänen, Feintuch, Hopkins, & Marantz, 2004), and concreteness (Kounios & Holcomb, 1994; Paller, Kutas, Shimamura, & Squire, 1988; West & Holcomb, 2000). The N400 can distinguish between syntactically or semantically defined word kinds, for example nouns and verbs (Federmeier, Segal, Lombrozo, & Kutas, 2000) or animal and tool names (Kiefer, 2001). It was proposed that an early part of the N400, called N350 or, in magnetic field recordings, M350, reflects lexical processing (Pylkkänen & Marantz, 2003), and can be separated from the N400 proper, which is best characterized as an index of semantic memory use during language comprehension and context integration (Kutas & Federmeier, 2000). N400-like responses are also known to reflect grammar processing or syntactic working memory operations (Bornkessel & Schlesewsky, 2006; Coulson, King, & Kutas, 1998; Kotz, Frisch, von Cramon, & Friederici, 2003; Münte, Heinze, & Mangun, 1993; Münte, Heinze, & Prevedel, 1990). Discourse context has also been shown to have an influence on the N400 (Nieuwland & Van Berkum, 2006; van Berkum, Hagoort, & Brown, 1999). Furthermore, it has been found to be modulated even by nonlinguistic stimuli such as pictures (Barrett & Rugg, 1990), sounds (Van Petten & Rheinfelder, 1995), and odours (Grigor, Van Toller, Behan, & Richardson, 1999).

The brain generators of the N400 components have not been unambiguously identified. The scalp topography of the N400 with its parietal negativity maximum may be generated in left fronto-temporal cortex (Salmelin & Kujala, 2006; Tse et al., 2007; Van Petten & Luka, 2006), but inferior-temporal and bilateral hippocampal sources have also been discussed (Curran, Tucker, Kutas, & Posner, 1993; Nobre & McCarthy, 1995).

An even later ERP component, the P600, is related to syntax and grammar processing. This P600 component can be elicited by a range of violations of grammar rules, including rules of phrase structure (“The man sold the *from* house”), agreement and subjacency. A P600 wave can even emerge to sentences that are grammatically correct, but in most individuals would first elicit a garden path effect associated with the temporary (but incorrect) impression that the string is ungrammatical (Hagoort et al., 1993; Osterhout & Holcomb, 1992; Osterhout, Holcomb, & Swinney, 1994; Tse et al., 2007).

Another ERP component sensitive to grammatical stimulus manipulations, the N125, has been reported for much earlier latencies between 100 and 250 ms. Because of this early latency and its characteristic topography (negativity at left-frontal electrodes), it has been named “Early Left Anterior Negativity” (ELAN, Friederici, Pfeifer, & Hahne, 1993; Neville, Nicol, Barss, Forster, & Garrett, 1991). It was first observed with violations of rules that bind syntactic constituents into tree structures (which, in classic syntactic theory, were called phrase structure rules). A similar early left-lateralized negativity was also found for grammatical function words (Neville, Mills, & Lawson, 1992; Nobre & McCarthy, 1994; Pulvermüller, Lutzenberger, & Birbaumer, 1995), and the same time range revealed differences in cortical activation between other lexical categories, too, for example between nouns and verbs (Brown & Lehmann, 1979; Brown, Lehmann, & Marsh, 1980; Dehaene, 1995; Preissl, Pulvermüller, Lutzenberger, & Birbaumer, 1995; Pulvermüller, Lutzenberger, & Preissl, 1999). It was therefore argued that the ELAN reflects an early stage of grammar processing where the information about a word’s lexical category is accessed and a phrase structure representation is being built (Friederici, 2002). An influential serial model states that this early access to lexico-syntactic structure is distinguished from later processing of thematic role and semantics (N400 and N400-like responses) and a terminal step of syntactic integration and re-processing (P600, Friederici, 2002).

The serial perspective on interpreting neurophysiological effects of psycholinguistic processing critically depends on the qualitative distinctness of neurophysiological effects and their specificity in the linguistic-cognitive domain (Bornkessel & Schlesewsky, 2006; Friederici, 2002). In this context, the distinction between an early and a late stage of syntactic analysis builds on the claim that only syntactic violations of a specific type (phrase structure violations) can lead to the syntactic ELAN. This stipulation was, however, called into question by the observation that syntactic processes outside the domain of tree-based syntactic binding relationships, especially agreement violations, are reflected in the early negativity (Barber & Carreiras, 2005; Deutsch & Bentin, 2001; Shtyrov, Pulvermüller, Näätänen, & Ilmoniemi, 2003). The early neurophysiological manifestation of agreement casts doubt on two-stage parsing models, as agreement processes would, according to these models, be subsequent to the build-up of syntactic tree structures. A recent suggestion is that latency differences between some syntactic brain responses have a methodological rather than genuinely syntactic origin (Hasting & Kotz, 2008; Pulvermüller & Shtyrov, 2006).

In sum, the serial/cascaded processing perspective emerging from these data is the following: after initial analysis of physical features of critical word stimuli, lexical category information is retrieved and an elementary syntactic structure is built (early left-anterior negativity). Subsequently, lexical processing and semantic access and context integration predominate (N400). Finally, there is an optional second step of in-depth syntactic analysis or re-parsing (P600, Fig. 1, Friederici, 2002). There are still issues about the functional distinctness of early and late syntactic stages.

## 3 Early near-simultaneity

### 3.1 Preconditions for obtaining early effects: methodological issues

In the following, we will review recent evidence for early (< 250 ms) physiological manifestations of psycholinguistic information processing during written and spoken language comprehension. Before we do this, a methodological issue must be addressed: The N400 and other late effects are large, robust and have been replicated in numerous experiments. In contrast, although early effects have been reported by a growing number of studies, they are usually small and different studies have sometimes produced inconsistent results. In the case of syntax, only a small number of studies supported the existence of early effects for some time (Friederici et al., 1993; Neville et al., 1991) while others could not document them (Hagoort et al., 1993; Osterhout & Holcomb, 1992). Only over time, agreement emerged about their existence. Similarly, Rugg’s initial report that lexicality, the difference between meaningful words and regular pseudowords, is first reflected in the N100–P200 complex (Rugg, 1983), and Bentin et al.’s pioneering observation of an early effect of semantic priming at ∼200 ms after visual word onset (Bentin, McCarthy, & Wood, 1985) contrast with a much larger number of reports of lexical and semantic information reflected in the N400 (see previous section). Why are early effects so evasive to the EEG and MEG measurements?

We would like to put forward two possible answers to this question: (1) Early effects are short-lived and focal, whereas late effects are long-lasting and widespread. Previous studies, if they looked at early latencies at all, usually employed rather large time windows (⩾100 ms) even for early ERP components, which might have obscured these short-lived effects. (2) Because early effects are smaller in amplitude and more focal in distribution, they are more sensitive to variance in stimulus parameters.

Support for point (1) is provided in the top and middle diagrams of Fig. 2. This figure illustrates significant effects of word frequency both on the N400 component and around 200 ms (Dambacher, Kliegl, Hofmann, & Jacobs, 2006). The latter effect is clearly smaller in duration (possibly a few tens of milliseconds) than the former (several hundreds of milliseconds), and might have been missed if a large time window had been chosen for analysis. Similar arguments have been presented previously for both visual and auditory stimuli (Pulvermüller, 1999; Pulvermüller & Shtyrov, 2006). The bottom diagram of Fig. 2 further illustrates point (2). In this case, effects of word frequency were found at different latencies for short and long words, in the event-related magnetic brain response measured with MEG (Assadollahi & Pulvermüller, 2001, 2003). Interestingly, the frequency effect for short words appeared very early, at 100–150 ms, and a general frequency effect was present at ∼200 ms. A later word frequency effect was primarily carried by longer words (Assadollahi & Pulvermüller, 2001, 2003). The earliest effect of word frequency is not detected if data are collapsed across short and long words – as might have been the case in previous studies that were not interested in effects of word length. Another example for the disappearance of early effects with stimulus variance has been provided by a study using an N400 paradigm: Penolazzi and colleagues found an interaction of cloze probability with word length at early latencies which contrasted with a cloze probability main effect in the N400 range (Penolazzi, Hauk, & Pulvermüller, 2007). These results show the same lethal effect of stimulus variance (here: in word length) on early brain responses indexing word frequency and semantic context. Exact matching of stimulus properties, also minimizing stimulus variance, and examining reasonably narrow time windows seem to be preconditions for obtaining early effects.^2^

### 3.2 Written language

As previously mentioned, psycholinguistic processes are reflected early by the syntactic ELAN (Friederici et al., 1993; Neville et al., 1991) and lexical-category responses (Brown & Lehmann, 1979; Brown et al., 1980; Neville et al., 1992). In one particular study, grammatical function words also elicited a left-lateralized component very similar in scalp topography to the syntactic negativity, with latencies as early as 160 milliseconds, whereas content words referring to objects and actions elicited a bilateral early negativity with the same latency (Pulvermüller et al., 1995). Words from different syntactic classes, especially nouns and verbs, and words differing in their meanings elicit different early responses in the event-related potential and magnetic field (Brown & Lehmann, 1979; Brown et al., 1980; Dehaene, 1995; Hauk & Pulvermüller, 2004b; Kellenbach, Wijers, Hovius, Mulder, & Mulder, 2002; Koenig & Lehmann, 1996; Preissl et al., 1995; Pulvermüller, Hummel, & Härle, 2001a; Pulvermüller et al., 2001b; Pulvermüller, Lutzenberger, & Preissl, 1999; Pulvermüller & Shtyrov, 2008). Although it had been argued that early differences between syntactic classes (noun/verb) could be explained on the basis of semantic properties (action-/visually-related semantic features, Pulvermüller, Mohr, & Schleichert, 1999), other authors found a lexico-syntactic effect independent of semantic differences (Kellenbach et al., 2002).

Further work documented early reflections of a range of psycholinguistic variables, including the frequency with which words occur in normal text (word frequency), word length, and typicality (the likelihood with which letters of a word occur together, Assadollahi & Pulvermüller, 2001; Hauk et al., 2006a, 2006b; Sereno, Rayner, & Posner, 1998). Word meaning (semantics) and even affective-emotional properties of words were reflected in early brain responses as well (Hauk & Pulvermüller, 2004b; Hinojosa, Martin-Loeches, & Rubia, 2001; Kiefer, Schuch, Schenck, & Fiedler, 2007; Kissler, Herbert, Winkler, & Junghofer, 2008; Scott, O’Donnell, Leuthold, & Sereno, 2008; Skrandies, 1998). For example, Hauk and Moscoso del Prado Martin found local activation differences to written words from different semantic categories matched for a range of psycholinguistic variables at ∼200 ms after stimulus onset (Hauk & Pulvermüller, 2004b; Moscoso Del Prado Martin, Hauk, & Pulvermüller, 2006). Slightly earlier, the first effects of word frequency were seen (Hauk & Pulvermüller, 2004a). In summary, it appears that all types of information bound to single written words, be they physical, orthographic, lexical or semantic in nature, are reflected by neurophysiological indicators within the first 200 ms after word onset.

**Fig. 3:**
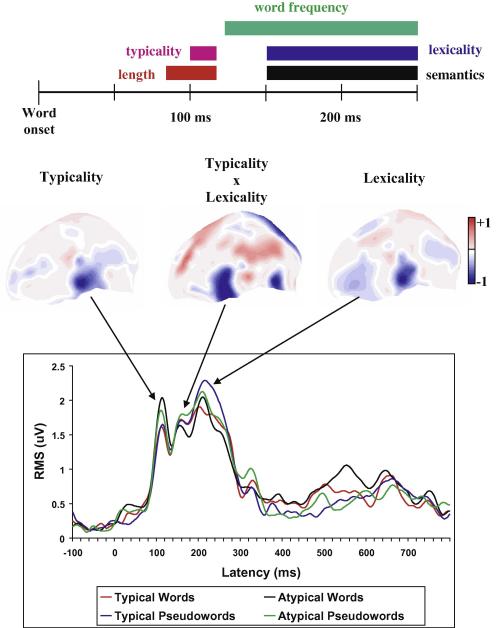
Near-simultaneous early brain-reflections of psycholinguistic information types in word reading. Top diagram: written words that differ in their form, frequency and meaning lead to different brain responses early-on. Features of the written word form (word length, typicality) tend to become manifest before lexico-semantic information (about word frequency, lexicality, semantics). This supports at least two stages in lexical processing, a form-related process at 100 ms and a lexico-semantic process at 150–200 ms (after Hauk et al., 2006a). Bottom diagram: Early processing stages related to form and meaning draw upon the same brain areas. The typicality effect at 100 ms was localized in posterior inferior-temporal cortex (in fusiform gyrus, see McCandliss et al., 2003) and the subsequent lexico-semantic effect involved the same area, consistent with its reactivation in lexico-semantic processing (see Price & Devlin, 2003) (modified from Hauk et al., 2006b).

But would context processing become manifest similarly early or only at a later stage? Sereno and collaborators were the first to report neurophysiological indexes of semantic context integration within a sentence before 200 milliseconds after onset of critical written words (Sereno, Brewer, & O’Donnell, 2003). Early effects of semantic congruency before and around 200 ms were confirmed and localized to temporal cortex using a new imaging technique, event-related optical signal (EROS) (Tse et al., 2007). Penolazzi and her colleagues further confirmed early semantic context effects by demonstrating that they critically depend on the length and frequency of written words (Penolazzi, Hauk, & Pulvermüller, 2007). This explains why previous studies did not find consistent early effects even when stimulus parameters were matched; variability of stimulus parameters may have abolished them. Taken together, these results argue in favor of early near-simultaneity of a wide range of psycholinguistic and cognitive processes in written language comprehension (Fig. 3).

### 3.3 Spoken language

When written language stimuli appear on a computer screen, the full information about their form and meaning is simultaneously present and latencies can therefore be computed relative to word onset. As speech unfolds in time, the computation of latencies and choice of zero points is more delicate for spoken language. If zero is at word onset, a word such as “locomotive”, which becomes distinct from its rival “locomotion” only at its 4th syllable onset, might receive unrealistically late physiological indexes of recognition latencies. Most studies reporting late neurophysiological responses, for example in the N400 to spoken words, calculated latencies relative to word onset (for example Halgren et al., 2002; Holcomb & Neville, 1990). To make studies of speech comparable with work on written language, it is essential to relate latencies to the point in time when the sensory information necessary for word recognition is available.

In psycholinguistics a distinction is made between the point in time within a word when it can first be unambiguously identified – the *uniqueness* or *isolation* point – and the earlier point were the word is usually first recognized with some confidence even though perceptual information is not yet fully sufficient for unique identification – the *recognition* point (Marslen-Wilson, 1987; Marslen-Wilson & Tyler, 1980). Interestingly, cortical signatures of word comprehension have been observed before the isolation point, indicating, in accordance with behavioral data (Marslen-Wilson, 1987; Zwitserlood, 1989), that the language comprehension system engages in comprehension and semantic processing on the basis of incomplete information (Hagoort & Brown, 2000; Müller & Kutas, 1996; Van Petten, Coulson, Rubin, Plante, & Parks, 1999). Recent results indicate that the latency of probabilistic word *recognition* is reflected in early MEG activity (100–150 ms after word recognition point, Pulvermüller, Shtyrov, Ilmoniemi, & Marslen-Wilson, 2006). A realistic zero point for calculating the latency of brain indexes of recognition and comprehension may therefore be the recognition point, as determined by behavioral experiments (Marslen-Wilson, 1987; Marslen-Wilson & Tyler, 1980).

However, due to the jitter of recognition points, realigning the time frame for data analysis does not resolve the problem that speech stimuli are naturally variable in their acoustic characteristics and recognition points. These problems can be avoided if brain responses are recorded to the same stimulus and this stimulus is presented in different contexts, where it elicits different psycholinguistic processes (Pulvermüller & Shtyrov, 2006). An example is the phoneme [t] which gives rise to word recognition in the context of “bite” and would lead to phonological processes but not to lexical access in the context of the pseudoword “pite”.

The Mismatch Negativity paradigm offers a unique opportunity to realize such designs. The critical stimulus is presented many times in an oddball sequence, where a frequent so-called standard stimulus (for example [paj]) is randomly replaced by one or more rare deviant stimuli (for example [pajt]). The deviant stimulus elicits the Mismatch Negativity, or MMN, which is calculated by subtracting the brain response of the standard from that of the deviant stimulus (that is, [pajt] minus [paj]). In the EEG, the MMN has a latency of 100–250 ms and a fronto-central maximum with major sources in superior-temporal cortex of both hemispheres (Näätänen, Gaillard, & Mäntysalo, 1978; Näätänen, Tervaniemi, Sussman, Paavilainen, & Winkler, 2001). Additional sources in fronto-central cortex may reflect stimulus characteristics and cortical circuits activated by specific stimuli.

The MMN is of particular interest for cognitive scientists, because its magnitude and sources reflect the activation of memory circuits in the brain that represent and process phonemes and spoken words (Naatanen, Paavilainen, Rinne, & Alho, 2007). For example, a sound that distinguishes between meaningful words in a language the subject is proficient in shows a stronger left superior-temporal MMN activation compared with similar but unfamiliar sounds (Dehaene-Lambertz, 1997; Näätänen et al., 1997; Winkler et al., 1999). If a syllable or phoneme is placed in the context of a meaningful lexical item, it elicits a stronger MMN activation in superior temporal cortex compared with the same speech stimulus presented in the context of a meaningless pseudoword (MMN to [t] in [bajt] larger than that in [pajt]) (Korpilahti, Krause, Holopainen, & Lang, 2001; Pettigrew et al., 2004; Pulvermüller et al., 2001b; Shtyrov, Pihko, & Pulvermüller, 2005; Shtyrov & Pulvermüller, 2002). The existence of cortical memory circuits at different levels (phonological, lexical) is therefore reflected in the neurophysiological response. The latencies of the relevant differences were within 200 ms after the critical phoneme, syllable or word could first be identified.

**Fig. 4:**
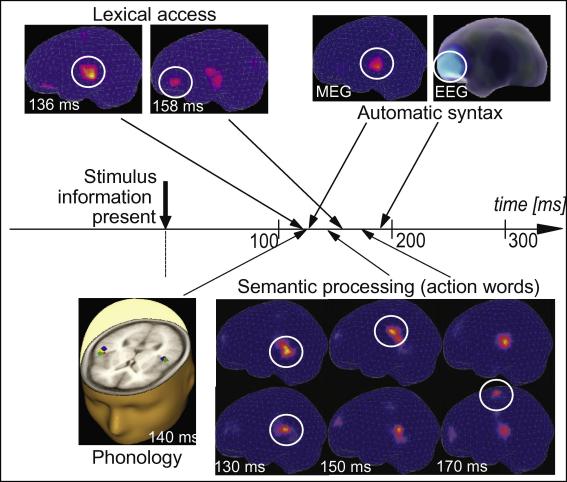
Near-simultaneous early brain-reflection of psycholinguistic information types in speech comprehension. On-line speech comprehension processes as studied using the mismatch negativity, MMN, a brain response to acoustic change detection also reflecting the activation of memory traces in the human brain (Näätänen et al., 2001). Phonological processing became manifest in a modulation of the MMN around 140 ms (Näätänen et al., 1997; Shtyrov, Pihko, & Pulvermüller, 2005) and lexicality was reflected by two sources in superior-temporal and inferior frontal cortex, sparked, respectively, at 136 and 158 ms (Pulvermüller et al., 2001, 2003). Syntactic violations elicited a syntactic MMN at about the same time, with sources in inferior frontal and superior-temporal cortex (Menning et al., 2005; Shtyrov et al., 2003). Semantic effects were seen at 140–170 ms when the same syllables were presented in words that indicated face/arm or leg actions (Pulvermüller et al., 2005). These results are consistent with near-simultaneous early access to different types of psycholinguistic information. Critically, there were fine-grained time lag differences, especially in the semantic domain: Leg-related words (e.g., “kick”) activated the central-dorsal sensorimotor leg representation 30 ms later than inferior-frontal areas were sparked by face/arm-related words (“eat”, “pick”). This shows category-specificity in the temporal structure of semantic brain activation.

Semantic effects could also be demonstrated in the early response, for example in the differential activation of motor areas elicited by action words referring to different parts of the body. It had been shown in previous studies using a range of experimental techniques that words semantically related to motor actions do activate the motor system when being perceived (Pulvermüller, 1999). Furthermore, this motor activation even reflects fine-grained referential meaning of the action-related words. Words regularly used to refer to actions performed with the legs, arms or face activate the pre-motor and motor cortex in a somatotopic manner (Hauk, Johnsrude, & Pulvermüller, 2004; Pulvermüller et al., 2001). A leg-related word such as “kick” activates dorsal areas, where leg actions are represented and processed, whereas arm-related words such as “pick” or face-related words such as “lick”, activate lateral or inferior frontal motor areas. The semantic somatotopy of action words documented by fMRI calls for neurophysiological research into the time course of category-specific semantic activation. MMN experiments have shown that motor regions are being sparked rapidly, within the first 140–170 ms after the recognition point of action-related words. There was also evidence for fine-grained latency differences, as face- and arm-words tended to activate inferior frontal cortex earlier than leg words activated the centro-dorsal leg region (Pulvermüller, Shtyrov, & Ilmoniemi, 2005; Shtyrov, Hauk, & Pulvermüller, 2004). Taken together, these results confirm near-simultaneous early brain correlates of phonological, lexical and semantic information immanent to a spoken word within the first ∼150 ms after the auditory input allows for word identification (Fig. 4).

Syntactic effects similar in both scalp topography and cortical generators to the syntactic early left-anterior negativity could also be revealed in the MMN response, with latencies similar to those of lexical and semantic MMN effects (Hasting & Kotz, 2008; Hasting, Kotz, & Friederici, 2007; Menning et al., 2005; Pulvermüller & Shtyrov, 2003; Shtyrov et al., 2003). Interestingly, early neurophysiological syntax effects are not explained by sequential probabilities of words in strings but rather indicate that discrete rule-like mechanisms are at work (Pulvermüller & Assadollahi, 2007). The attention-independence of early syntax effects could be demonstrated by orthogonally varying grammaticality and task parameters (Hahne & Friederici, 1999; Pulvermüller, Shtyrov, Hasting, & Carlyon, 2008). According to recent work, also semantic context integration is reflected by the MMN at early latencies (Menning et al., 2005; Shtyrov & Pulvermüller, 2007). Neurophysiological studies of spoken language had earlier shown that semantic expectancy violation at the level of discourse and text processing can also lead to early manifestations in the event-related brain potential (Brown, van Berkum, & Hagoort, 2000). These results are consistent with the early context integration effects seen for written words in sentence context.

## 4 Interim summary

The first brain processes indexing lexical, semantic and syntactic processes occur early, within 200–250 ms after stimulus information allows for unambiguous identification of critical language elements (Figs. 3 and 4). As phonological and orthographic information is also brain-reflected in the same time range (Eulitz, Diesch, Pantev, Hampson, & Elbert, 1995; Holcomb & Grainger, 2007; Petit, Midgley, Holcomb, & Grainger, 2006; Shtyrov, Kujala, Palva, Ilmoniemi, & Näätänen, 2000), this early near-simultaneity appears consistent with parallel models of language and cognitive processing, at least on first view. Because the late responses (N400, P600), which start after 200 ms, are preceded by substantially earlier indicators of the same types of stimulus information, they are unlikely to reflect the first, initial stages of information access. Because of their precursors, they must reflect secondary processes following early syntactic, lexical or semantic information access and context integration. It is possible that processing at later stages is necessary for parsing and understanding a meaningful sentence and that it is these stages of the comprehension process that are reflected by long-latency event-related neurophysiological responses. A different possibility is that comprehension processes terminate early, within 200 ms, and late brain responses reflect post-comprehension processes (Glenberg & Kaschak, 2002) specifically triggered by failure of semantic or syntactic integration of a word or other meaningful stimulus into its context. A range of post-comprehension processes may be relevant here, for example the use of semantic memory in predicting and pre-activating memory circuits of forthcoming words or for building a memory representation for the encountered stimulus (Kutas & Federmeier, 2000). However, also attempts to reprocess, revise and correct the “inaccurate” word, phrase or larger parse are potentially signified by late components (cf. Friederici, 2002).

The data summarized so far can be integrated as follows. Access to phonological, lexical and semantic word features along with semantic and syntactic context integration and parsing are early near-simultaneous processes reflected by brain responses with latencies between 100 and 200 ms. N400 and P600 might reflect a second step in lexical, semantic and syntactic information processing, or might, instead, indicate specific linguistic or non-linguistic post-comprehension processes.

In order to draw conclusions from neurophysiological data on serial or parallel models of language processing and comprehension, it is important to now consider what “early near-simultaneity” exactly means. Does it mean synchrony with millisecond precision or are there still minimal but well-defined time delays, although on a much finer time-scale than previously thought? To answer this question, it is necessary to consider additional experiments in which recognition latencies were exactly controlled, stimulus variance was minimized and different types of psycholinguistic information processing were compared in the same study.

## 5 Cortical processing steps and millisecond delays

In principle, a parallel approach to cognitive and language processing is consistent with the near-simultaneity of EEG/MEG signs of psycholinguistic information access in the first 200–250 ms. This near-simultaneity may be due to discrete cortical activation processes lasting approximately 1/5 s, during which all types of cognitive information related to a sign are accessed.

There is independent evidence across modalities that the interval of approximately 200 ms represents a basic discrete time step in cortical function. Auditory perceptual processes are integrated within a temporal window of ∼200 ms width (Eddins & Green, 1995; Green, Birdsall, & Tanner, 1957) and such perceptual integration is reflected by cortical brain activity (Tervaniemi, Saarinen, Paavilainen, Danilova, & Näätänen, 1994; Yabe et al., 1998). In the motor domain, tracking of a continuously moving target is done in discrete motor movements each lasting approximately 150–200 ms (Stark, 1968), and a universal feature of speech is that syllables are being produced with a frequency of up to approximately 5 Hz (Keller, 1990). This evidence converges on an a priori brain-pace of ∼5 processing steps per second.

The mechanistic basis of a discrete processing step of ∼200 ms in perceptual, motor and cognitive processes can be specified in neuronal and cognitive terms (MacKay, 1987). It has long been noted that learning and synaptic strengthening in an associative memory structured similar to the cortex must lead to very strongly connected neuronal circuits whose activation takes the shape of “mini-explosions”, so-called “ignitions”, interrupted by regulatory inhibition (Braitenberg, 1978; Garagnani, Wennekers, & Pulvermüller, 2008; Hebb, 1949; Palm, 1990; Willshaw, Buneman, & Longuet-Higgins, 1969). Such processing in discrete steps may lead to a structuring of cognitive and language processes into temporal chunks (Braitenberg & Pulvermüller, 1992; Wennekers, Garagnani, & Pulvermüller, 2006). In this model, the early near-simultaneity of neurophysiological indexes of psycholinguistic information access is based on the ignition of neuronal circuits binding this information to the incoming sign.

Even though discrete processing steps of 1/5 s may be relevant in cortical function, brain physiology operates on a millisecond time scale. The lag with which sensory stimuli elicit the first neuronal activation in primary auditory and visual cortex is in the range of 20–90 ms, as shown by both invasive and non-invasive studies in monkeys and humans (Di Russo, Martinez, & Hillyard, 2003; Godey, Schwartz, de Graaf, Chauvel, & Liegeois-Chauvel, 2001; Hubel, 1995; Lütkenhöner et al., 2003; Maunsell & Gibson, 1992; Pelizzone et al., 1987; Schroeder & Foxe, 2002). Conduction times between distant cortical areas can, as far as the most frequent myelinated axons are concerned, be estimated to lie in the range of 10–50 ms (Aboitiz, Scheibel, Fisher, & Zaidel, 1992; Miller, 1996). Direct measurements using MEG (Pulvermüller & Shtyrov, 2009; Pulvermüller, Shtyrov, & Ilmoniemi, 2003) and cortical stimulation and simultaneous subdural recording (Matsumoto et al., 2004, 2007) confirm such fast cortico-cortical activity conduction. To understand the brain processes of language at the mechanistic level of nerve cell circuits, it is essential to ask whether *near*-simultaneity of early brain processes indexing language implies strict synchrony or might still allow for fine-grained differences between activation times.

Certain speech stimuli, for example the chirp-like noises constituting stop consonants, are perceived as bare noise if placed out of speech context or in the context of other noise stimuli. However, they are perceived as phonemes if presented in appropriate speech context. These speech sounds can have the role of affixes and meaningful language units, morphemes, so that their context not only determines whether they are perceived as noise or speech but also whether they carry meaning or not. Presented in noise, phonemic and morphemic conditions, such critical identical stimuli elicited specific brain responses, with strongest left-laterality in the morphemic condition (Shtyrov, Pihko, & Pulvermüller, 2005). Remarkably, the latency of the brain response (136–155 ms) did not differ significantly between noise, phonemic and morphemic conditions. This result is consistent with strict simultaneity and parallelism of acoustic, phonological and lexical information access in speech processing.

In written word processing, small delays have been observed between the brain indicators of physical and higher linguistic-conceptual processes (word length vs. lexical access, Assadollahi & Pulvermüller, 2001; Hauk & Pulvermüller, 2004a; Sereno et al., 1998). In one study exploiting multiple regression analysis to disclose relationships between the magnitude of ERPs and psycholinguistic variables, there was an early reflection of word length and typicality (∼100 ms), followed by indexes of lexical and semantic processing (∼150 ms, Hauk et al., 2006a). This suggests a 20–50 ms delay between processing onset of written word form and lexico-semantic information. Similar results were obtained using a factorial design (Hauk et al., 2006b). Interestingly, the earliest effect related to the level of word form processing was present in inferior-temporal cortex, whereas the 150 ms effects of lexicality and semantics were seen in a more distributed set of areas including also perisylvian cortex. Around 200 ms, additional lexicality effects arose in inferior-temporal areas, consistent with reactivation of this area (Fig. 3).

Fine-grained differences in cortical activation times were seen between lexical brain processes (Pulvermüller & Shtyrov, 2008; Pulvermüller et al., 2003). The first brain responses distinguishing between spoken words and pseudowords arose around 130 ms after the critical stimulus information was present. The word-evoked activation pattern, however, included an activation source in superior-temporal cortex closely followed by activation in inferior-frontal cortex. The delay measured using MEG and source localization, 10–30 ms, is in good agreement with direct measurements from the cortex after direct stimulation (Matsumoto et al., 2004,2007). The second inferior-frontal generator was sparked at the same time (140–150 ms) when also meaning-related brain activation emerges (Pulvermüller et al., 2005).

When MEG and EEG were used to investigate the physiological basis of semantic comprehension processes, the action-word-induced activation of specific parts of the sensorimotor cortex revealed different activation times. As mentioned, the leg-area activation to leg-related words was found to be slightly delayed relative to the inferior-frontal face- and arm-related semantic activation (Pulvermüller et al., 2001a, 2005; Shtyrov et al., 2004). That these activations were manifestations of semantic word features was confirmed by a significant correlation between semantic ratings of stimulus words and the magnitude of local activation in leg- and face-regions (Pulvermüller et al., 2005). As different types of semantic information were brain-reflected at different latencies, these results argue in favor of distinct category-specific semantic systems with different brain loci (Shallice, 1988; Warrington & McCarthy, 1983) and activation times.

In summary, in spite of near-simultaneity of early brain responses reflecting access to different linguistic information types, there are reliable fine-grained delays. One may argue that these delays could be explained by serial or cascaded models operating at a finer time scale than previously stated, with tens rather than hundreds of milliseconds as the relevant temporal grain size. Much of the available neurophysiological evidence is compatible with abstract cascaded interactive models (Dell, 1986; Dell et al., 1997; MacKay, 1987) if the activation delays between phonological/orthographic, lexical, syntactic and semantic “nodes” are assumed to be short. It is, however, equally important to consider the facts any fast cascading (or seriality) in a standard psycholinguistic model would leave unexplained. These include the following features, which we will focus on in the subsequent section:

1. *Synchrony* of the neurophysiological indicators of acoustic, phonological and lexical processing in superior temporal cortex along with differences in local activation strengths and distributed spatio-temporal activation patterns.
2. *Reactivation of the same cortical region*, posterior inferior-temporal cortex, *with minimal delay reflecting different psycholinguistic processes*, written word form and lexico-semantic access.
3. *Delay differences between area activations reflecting lexical and category-specific semantic processes*, in the motor system.

## 6 Topography and conduction delays in linguistic memory circuits as the key

To provide an account of early near-simultaneity and the critical latency differences between cortical area activations, a model is necessary that spells out language and conceptual processes in terms of mechanistic neuronal circuits and their activation. Mechanistic brain-based models of language are available since the early 1990 s (Braitenberg & Pulvermüller, 1992; Damasio, 1990; Feldman, 2006; Mesulam, 1990; Pulvermüller & Preissl, 1991). We first highlight here an account that postulates lexico-semantic circuits with specific cortical distributions (Pulvermüller, 1999, 2005; Pulvermüller and Preissl, 1991) and then briefly address the question how alternative approaches would address critical issues, paying special attention to the previously unexplained features listed at the end of the previous section.

The model posits that strongly connected neuronal ensembles spread out over different sets of areas of the cortex are the basis of cognitive and language processing. The momentary explosion-like ignition of one of these circuits accounts for near-simultaneity of area-specific activations and the conduction delays of cortical fibers within the circuits explain fine-grained activation delays in the millisecond range.

**Fig. 5:**
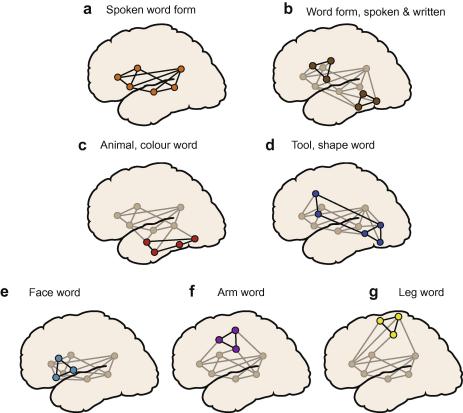
Lexical and semantic representations modelled at the mechanistic level of cortical circuits. As word learning implies linking spoken word forms to their respective articulatory patterns, the abstract articulatory acoustic pattern of a spoken word form is stored by strongly connected lexical circuits distributed over superior-temporal and inferior-frontal cortex (perisylvian cell assembly, a) In literate speakers, information about writing gestures and written word forms are bound to spoken word form representations; this binding of knowledge is cortically grounded in a halo of perisylvian cell assemblies extending into hand-related motor and premotor areas and fusiform gyrus (b). Meaningful words bind, in an arbitrary manner, information about their form and the concepts they refer to. Abstract semantic links are realized by the multiple connections between perisylvian cell assemblies and modality-specific semantic circuits in various parts of the cortex, for example in anterior- and inferior-temporal cortex (animal and color concepts, c), posterior-inferior and middle temporal cortex (tools and shapes, d), inferior-frontal cortex (face and articulatory actions, e), dorso-lateral fronto-central cortex (arm actions, f), and dorsal central cortex (leg actions, g). Ignition of these networks upon stimulation accounts for early near-simultaneity of neurocognitive indexes of psycholinguistic information access and conduction delays through long-distance cortico-cortical connections within these circuits explain fine-grained activation delays.

Neuronal circuits processing spoken word forms comprise neurons in superior-temporal cortex activated by phonetic features of a spoken word, neurons in inferior-frontal cortex programming and controlling articulatory movements and additional neurons connecting the acoustic and articulatory populations. Such a distributed fronto-temporal circuit, in perisylvian cortex, is shown in Fig. 5a. The strict simultaneity of acoustic, phonological and lexical processing indexes is explained by this model, as neuronal populations in the same local structure, in superior-temporal cortex, are assumed to contribute to acoustic, phonological and lexical processes. Therefore, conduction times of the auditory input to these critical sites are roughly the same.^3^ The superior-temporal lobe indeed seems to contribute to all of these processes (Scott, 2005; Uppenkamp, Johnsrude, Norris, Marslen-Wilson, & Patterson, 2006) and the local activation differences between noise, phonemes and speech revealed by fMRI may be explained, in part, by differential linkage to articulatory circuits. Importantly, the evidence for stronger cortical activation (Pulvermüller et al., 2001) and motor links (Fadiga, Craighero, Buccino, & Rizzolatti, 2002; Watkins, Strafella, & Paus, 2003) of words compared with pseudowords supports the existence of action-perception circuits for spoken words. Further evidence that these lexical memory networks link superior-temporal (acoustic) circuits to inferior-frontal (speech motor planning) circuits comes from imaging work revealing coactivation of these areas in speech processing (Pulvermüller et al., 2003, 2006). The frontal areas involved are sparked 10–30 ms after superior-temporal activation (Pulvermüller et al., 2003), which is, as mentioned, consistent with direct measurements of conduction inter-area delays in cortex (Matsumoto et al., 2004). Note that a classic psycholinguistic model of a mixed parallel-and-cascaded nature could possibly be tailored to capture the experimental facts summarized; it would, however, not provide a priori predictions on the cortical areas involved and, critically, the time delay between area activations and psycholinguistic sub-processes.

Representations of words as written forms and writing gestures are assumed to be linked to spoken word form representations via bi-directional connections. This extended fronto-temporal network sketched in Fig. 5b may underlie abstract, or transmodal, word form processing. The visual word form representation includes critical neurons in posterior inferior-temporal, especially fusiform cortex (McCandliss, Cohen, & Dehaene, 2003). However, posterior inferior-temporal neurons are also activated in visual object processing. Therefore, when a word is bound to an object representation, or, more generally, to specific visual-semantic features, the word form circuit will also be linked to inferior-temporal semantic circuits (Martin, Haxby, Lalonde, Wiggs, & Ungerleider, 1995; Moscoso Del Prado Martin et al., 2006; Pulvermüller & Hauk, 2006; Simmons et al., 2007). Neuronal links between word form representations and visual semantic-conceptual networks are illustrated in Fig. 5c and d.

Therefore, visual word recognition is modeled as early bottom-up processing in lexico-orthographic circuits (Fig. 5b) leading to lexico-semantic network activation in perisylvian cortex, which, in turn, entails “top–down” semantic activation in anterior-temporal and, finally, posterior inferior-temporal cortex (Fig. 5c and d). In the experimental tests, typicality, i.e., the frequency with which letter pairs and triplets occur together, and lexicality of written stimuli were varied to modulate the processing load on the orthographic and lexico-semantic circuits, respectively. At 100 ms, inferior-temporal activation reflected typicality, and at ∼200 ms, activation of the same area related to the lexicality feature (Fig. 3, bottom diagram, Hauk et al., 2006a, 2006b). This explanation of inferior-temporal reactivation capitalizes on the postulate that word form and semantic circuits involve the same area in posterior inferior-temporal cortex, a feature a classic cascaded psycholinguistic model without a brain basis would not capture.

To any box-and-arrow diagram with a single semantic centre, the differential cortical activations related to category-specific semantic processing and especially their different time lags constitute a major challenge. The neuronal circuit model in Fig. 5 explains such differences in terms of conduction delays within neuronal assemblies. Semantic circuits for action-related words connect lexical representations in perisylvian cortex with referential semantic information. These circuits reach into motor cortex and also control motor functions of the face, arm and leg, respectively (Pulvermüller, 2005). Because of the different neuroanatomical distributions of the circuits, it is conceivable that different time demands arise for activity to travel the short distance from perisylvian cortex to face- or arm-action representations as compared with the longer distance from the same perisylvian networks to the dorsal leg representation (Fig. 5e–g). Therefore, the mechanistic model provides an account of semantic category-specificity that is manifest in focal cortical activation and, critically, in the time course of semantic brain activation distinguishing between word kinds with different referential meaning.

## 7 Specific spatio-temporal patterns of activation: implications for brain theories of language

We here discuss brain models of language in the light of specific rapid activation spreading indexing different kinds of psycholinguistic processes. For models in the tradition of the established Lichtheim-Wernicke scheme (Lichtheim, 1885), where centers for speech production and comprehension are thought to be partly autonomous, the rapid activation of inferior-frontal cortex even in unattended speech processing appears as a stumbling stone. In comprehension, Lichtheim postulates “contact” between the centres for sound images and his concept centre, but not an involvement of the brain parts relevant for production or action (p. 207), especially inferior-frontal cortex. However, the growing evidence for rapid activation spreading from superior-temporal “receptive” language areas to inferior-frontal “production” areas in lexical and phonological processing (see Pulvermüller & Shtyrov, 2009; Pulvermüller et al., 2006; Fadiga et al., 2002; Watkins et al., 2003) and the evidence for a specific role of inferior-frontal and fronto-central motor areas in language comprehension (Bak, O’Donovan, Xuereb, Boniface, & Hodges, 2001; Bak et al., 2006; D’Ausilio et al., 2009) strongly argue against this modular perspective. A revision of the classic model is necessary that accounts for mirror neuron activation in language comprehension, a step taken by the present and related neuroscience-based models of language and conceptual processing (Fadiga & Craighero, 2006; Kemmerer & Gonzalez-Castillo, 2008; Pulvermüller, 1999, 2005; Rizzolatti & Craighero, 2004; Rizzolatti, Fogassi, & Gallese, 2001).

Connections between temporal and fronto-central language areas are by way of cortical fiber systems, especially the dorsal arcuate fascicle and the ventrally placed extreme capsule and uncinate fascicle, which respectively connect more posterior/lateral and anterior areas of the superior-temporal cortex to the inferior-frontal lobe (Glasser & Rilling, 2008; Makris et al., 1999; Romanski et al., 1999; Saur et al., 2008). These systems may carry different types of information in auditory and linguistic processing (Rauschecker & Tian, 2000; Scott & Johnsrude, 2003). In one view, the interface from speech to conceptual information is carried by the ventral stream (especially posterior lateral-temporal cortex) and the acoustic-phonetic-to-motor-articulatory link to the dorsal stream (posterior area Spt in the planum temporal and inferiorfrontal areas, Hickok & Poeppel, 2007). However, this position is in conflict with data on action-related language, especially activation of specific areas in the motor system reflecting *semantics* at the single word and sentence levels (Pulvermüller, 2005). These data show that the dorsal stream is not restricted to phonological processing, but plays a role in category-specific semantics, too (cf. Fig. 5, bottom diagrams). Likewise, phonological processes do not seem to be restricted to the dorsal stream, as imaging studies show speech-specific activations in anterior *and* posterior superior-temporal cortex (Scott & Johnsrude, 2003; Scott & Wise, 2004; Spitsyna, Warren, Scott, Turkheimer, & Wise, 2006) and, critically, phoneme-specific activations at *anterior* superior-temporal loci (Obleser et al., 2006; Pulvermüller et al., 2006). In sum, a strict dedication of phonological and semantic processing to dorsal and ventral streams of auditory processing, respectively, as suggested by Hickok and Poeppel is difficult to maintain. Anterior and posterior streams of auditory processing both appear to contribute to both phonological and category-specific semantic processing (Garagnani et al., 2008). However, for some action-related semantic networks the dorsal stream is of greater relevance, whereas object semantics draws more heavily on visually-related ventral streams (Fig. 5).

Models placing semantic binding in a single unitary cortical system – be it, for example, the entirety of the perisylvian cortex (Tyler & Moss, 2001) or a more focal area (e.g., lexical interface in posterior inferior-temporal cortex, Hickok & Poeppel, 2007), do not explain category-specific cortical activation in general or the spatiotemporal patterns of activity indexing semantic types in particular. However, category-models have difficulty explaining the steep decline of semantic knowledge with degradation of specific areas, most notably the temporal poles (Hodges & Patterson, 2007). A solution is offered by models integrating a “semantic hub” with category-specific distributed neural systems. Such a model has recently been proposed (Patterson, Nestor, & Rogers, 2007) and marrying it more closely with the category circuits of Fig. 5 may lead to an integrated account of both category-specific cortical activations along with specific and more global neuropsychological semantic deficits. In a similar vein, a combined model of “embodied” action-perception-related circuits plus symbolic networks (Mahon & Caramazza, 2008) is, in principle, compatible with the results summarized in this review.

Although the present results suggest a compression of the time scale at which psycholinguistic information is first accessed in the brain, down to a grain size explainable by conduction delays of the most common axon types in human cortex, they are largely compatible with stepwise access to lexical, syntactic and semantic information. A new feature is the suggestion that lexical and phonological information is accessed in the staggered ignition of superior-temporal and inferior-frontal neuron populations, and that semantic subsystems are likewise accessed at latencies determined by their cortical loci. As TMS work shows influences of motor systems activation on lexical and phonological processing (Meister, Wilson, Deblieck, Wu, & Iacoboni, 2007; Pulvermüller, Hauk, Nikulin, & Ilmoniemi, 2005), it appears that, apart from perceptual “bottom up” activation, “top down” activation from the semantic and phonological action networks are effective in language understanding. This argues for interactive models.

Recent proposals about the time course of psycholinguistic information access in language comprehension (Friederici, 2002; Holcomb & Grainger, 2007) can be adjusted to cover the present results by (1) changing the temporal grain-size of the processing onsets of different information types from the 100 ms-range (Fig. 1, left) to step sizes varying between 0 and 50 ms (Figs. 3 and 4), (2) allowing for multiple area activations per information type to account for the many-many relationship between psycholinguistic and brain processes (Fig. 5), and (3) splitting phonological as well as semantic processes into more specific subtypes. In addition, to explain millisecond delays in activation between areas and stimulus types, it seems advantageous to recur to neurocognitive (rather than abstract cognitive) concepts, such as that of synfire chains mechanistically implementing delay lines in a neurobiologically plausible manner (Shmiel et al., 2005).

## 8 Summary

The well-known late brain indexes of psycholinguistic information access and context integration are preceded by early near-simultaneous activity. This suggests that the brain analyses signs, their meanings and context relationships within already 100–250 ms after the sensory information about the identity of a critical stimulus is available. This observation falsifies serial and cascaded psycholinguistic models locating the earliest semantic processes at ∼400 ms and is consistent with parallel models postulating near-simultaneous access to all psycholinguistic information types. However, there are fine-grained but reliable millisecond delays between cortical area activations in language processing that are not explained by parallel models, but require serial or cascaded processing on a much finer time scale than standard serial models propose. We argue that, in order to explain the range of locally specific activation delays, it is not sufficient to attach cortical area labels to standard psycholinguistic models. A neurobiological model specifically linking signs and symbols to neuronal circuits distributed over specific sets of cortical areas offers an explanation of recent results about the millisecond activation time course, including three critical observations: (i) strict simultaneity of phonological and lexical activation in superior-temporal cortex in spoken word processing, (ii) reactivation in posterior inferior-temporal cortex indexing form-related and lexico-semantic activations in written word recognition, and (iii) different areas and activation time courses reflecting lexical and word-category-specific semantic processing.

## 9 Future trends and directions

As many psycholinguistic and conceptual processes seem to be brain-reflected both early and late, it is important to clarify the relation between the two. Are the early semantic, syntactic and lexical effects just the beginning of late effects? Differential dynamics such as the ones summarized in Fig. 3 speak against this possibility. Would the late processes just repeat the early ones, or occur only if the early ones are unsuccessful? Here, our own data indicate that the early near-simultaneous processes exhibit surprising specificity to information types, both topographically and in terms of cortical generators, whereas the late ones, for example the N400, seem equally modulated by different linguistic features (including word frequency, lexicality, and semantic properties, Hauk et al., 2006a, 2006b; Pulvermüller & Shtyrov, 2006). Still, are the late components reflections of prolonged specifically linguistic processes, in-depth- or re-processing, or would they rather reflect post-comprehension processes (Glenberg & Kaschak, 2002) following completed psycholinguistic information access and context integration? And how fixed are the lags anyway? Kutas and colleagues have recently shown that stimulus context can modulate the time lag of brain responses indexing word and object processing (Barber & Kutas, 2007). Such context dependence and flexibility is of greatest relevance in the study of cognitive processes and points the way to fruitful future research. The relationship between specific activation times of defined brain areas on the one hand and specific cognitive processes on the other is one of the most exciting topics in cognitive neuroscience at present. Addressing this issue using MEG/EEG and source localization has only become possible very recently. If successful, the new available methods will make it possible to read the activation signatures of cortical circuits processing language and concepts in the human brain in more and more detail, therefore propelling the science of behavioral-physiological correlation into a new era.

## Footnotes

1 Strictly serial models imply that the processes in one component are finished before the next starts to operate (Fodor, 1983; Friederici, 2002). However, most current serial models relax this requirement, thus allowing for processing cascades and partial temporal overlap in processing at different stages (Dell, 1986; Dell, Schwartz, Martin, Saffran, & Gagnon, 1997; Hagoort, 2008; Norris, McQueen, & Cutler, 2000). Both variants, non-overlapping serial and cascaded, have in common that the *onsets of the processes at different levels* are in sequence. We focus on serial processing onset here, and therefore include cascaded models when referring to serial ones. Note that the question of seriality vs. parallelism is orthogonal to that of modularism vs. interactionism and that of localist and distributed representations (Page, 2000).

2 We further elaborate on methodological issues in the Section 5.

3 Note that even more sensitive methods may still find micro-delays of ∼10 ms and below possibly reflecting sub-regional activation differences within the greater superior-temporal area.
